# 

**Published:** 1874-04

**Authors:** 


### TABLE OF CONTENTS.

ORIGINAL COMMUNICATIONS.

Thoughts Upon Prescribing. By Frank A. Ramsey, M.D. - - - 191
Senile Hyperthrophy of the Prostate, with cases. By James S. Bailey, M.D. 205

CORRESPONDENCE.

Letter from L. R. Lincecum, M.D., of Long Point, Texas - - - 217
Letter from B. P. Reese, M.D., of Stanton, Va. - - - - - - 219
Letter from W. A. Cusick, M.D., of Gervais, Oregon - - - - - 220
Earth Poultices. By J. H. Payne, M.D., of Preston, Miss. - - - 221

REMARKS, GLEANINGS AND EXTRACTS.

Coffee an Antidote for Morphia; Chloroform—223. The Way to Increase the Curative Power of Iodide of Potassium; Tenia Removed by Pumpkin Seed; Sewing Machines Condemned; Retention of Urine—224. Carbolic Acid in Diabetes Mellitus; Child Murder; Trichinæ; Graduates; A Remedy for Headache; Intussusception; Neurosis and Neuralgia; Syphilitic Neuralgia; Gonorrhœa—225. By A. R. Kilpatrick, M.D.

Laws of Congenital Syphilis—226. The Local Treatment of Cavities in the Lungs; Ovarian Hernia—227. Epilepsy Caused by a Needle in the Ear; Treatment of Enuresis; Congenital Syphilis in Six Successive Infants; Lotion for Fœtid Feet—228. Silicate of Soda in Vesical Disease; Removal of Five Inches of the Sciatic Nerve; Mass of Scybala Mistaken for an Ovarian Tumor; First Carotid Ligation in the West; Inoffensive Sponge-Tents—229. By T. Curtis Smith, M.D.

The Therapeutical Value of the Sulphides—230. Spinal Anæmia—232. Acute Articular Rheumatism—233. Treatment of Acute Rheumatism by Means of Firm Dressing; Use of Chlorate of Potash and Glycerine Injections for the Ulcerations in Chronic Dysentery—234. Collodion and Silk Gauze as a Dressing in Mammary Abscess; French Experiments with Oatmeal—235. Uses of Conium; Inhalations of Muriate of Ammonia in Chronic Affections of the Chest; A Simple Plug for Rectal Hemorrhage—236. Albuminuria; Bromide Potassium as a Diuretic; Sir James Paget—237. Acetic Acid in Psoriasis; How to Check Coughs; Hemorrhoids in Children; Cholera Infantum—238. An Old Cancer Cure Revived; Administration of Ether; Tincture of Iodide of Tannin of Dr. Boinet; Remedy for Chronic Hoarseness; Syrupus Antiasthmaticus; Churchill's Tincture of Iodine—239. Treatment of Biliary Calculus by Choleate of Soda; Cancrum Oris Treated by Saturated Solution of Iodine; The Witch Hazel as a Hæmostatic; Nocturnal Muscular Cramps; Worm Remedy; Aqua Crystallina; Asthmatic Pastiles—240. Sulphur in Diphtheria; Galega Officinalis as a Lactogenetic; Dr. Tobias' Venetian Horse Liniment; Sinapisms; Phosphorus Mixture; Cough Mixture in Consumption—241. By Local Editors.

EDITORIAL AND MISCELLANEOUS.

Notices - - - - - - - - - - 242
A Convention of Confederate Surgeons; Removal of Minnie-Ball from Pelvis; The Virginia Medical Monthly - - - - - - - 243
Communications - - - - - - - - - 244
Advertising Department; Dr. G. H. Hodge; Normal Ovariotomy - - 245
The Rewards of Science - - - - - - - 246
Bibliographical - - - - - - - - - 247
Motion - - - - - - - - - - 252

